# Co_3_O_4_ Supraparticle‐Based Bubble Nanofiber and Bubble Nanosheet with Remarkable Electrochemical Performance

**DOI:** 10.1002/advs.201900107

**Published:** 2019-04-15

**Authors:** Jun Huang, Yingbo Xiao, Zhongyou Peng, Yazhou Xu, Longbin Li, Licheng Tan, Kai Yuan, Yiwang Chen

**Affiliations:** ^1^ College of Chemistry/Institute of Polymers and Energy Chemistry (IPEC) Nanchang University 999 Xuefu Avenue Nanchang 330031 China

**Keywords:** bubble nanofibers, bubble nanosheets, Co_3_O_4_ supraparticles, supercapacitors

## Abstract

Hollow nanostructures based on transition metal oxides (TMOs) with high surface‐to‐volumetric ratio, low density, and high loading capacity have received great attention for energy‐related applications. However, the controllable fabrication of hybrid TMO‐based hollow nanostructures in a simple and scalable manner remains challenging. Herein, a simple and scalable strategy is used to prepare hierarchical carbon nanofiber (CNF)‐based bubble‐nanofiber‐structured and reduced graphene oxide (RGO)‐based bubble‐nanosheet‐structured Co_3_O_4_ hollow supraparticle (HSP) composites (denoted as CNF/HSP‐Co_3_O_4_ and RGO/HSP‐Co_3_O_4_, respectively) by solution self‐assembly of ultrasmall Co_3_O_4_ nanoparticles (NPs) assisting with polydopamine (PDA) modification. It is proved that the electrochemical performance of Co_3_O_4_ NPs can be greatly enhanced by the rationally designed nanostructure of bubble‐like supraparticles combined with carbon materials as excellent electrodes for supercapacitors. The favorable structure and composition endow the hybrid electrode with high specific capacitance (1435 F g^−1^/1360 F g^−1^ at 1 A g^−1^/5 mV s^−1^) as well as fantastic rate capability. The asymmetric supercapacitors achieve an excellent maximum energy density of 51 W h kg^−1^ and superb electrochemical stability (92.3% retention after 10 000 cycles). This work suggests that the rational design of electrode materials with bubble‐like superstructures provides an opportunity for achieving high‐performance electrode materials for advanced energy storage devices.

## 1 Introduction

With the rapid development of electrical vehicles, portable electronic devices, and stationary grid storage, methods of exploiting efficient, safe, and sustainable energy storage devices are urgently needed.^1^, ^2^, ^3^ Supercapacitors (SCs), which can shorten the big gap of power density and energy density between conventional capacitors and batteries, have high potential for application in many areas.^4^, ^5^ However, it is hard for the limited energy density of carbon‐based electrochemical double‐layer capacitors to meet the requirements of large‐scale energy applications.^6^ Much effort has been dedicated to discover alternative materials to increase the energy density of SCs.^7^, ^8^, ^9^ Among them, transition metal oxides (TMOs; such as RuO_2_, NiO, CoO*_x_*, MnO_2_, and Fe_2_O_3_) have attracted considerable attention owing to their high theoretical specific capacitance, environment friendliness, and low cost.^10^, ^11^, ^12^ In particular, Co_3_O_4_ has received significant interest as electrode material for SCs, and it is considered to be a potential candidate for state‐of‐the‐art RuO_2_.^13^, ^14^ Unfortunately, the sluggish reaction kinetics and poor conductivity cause a unsatisfactory capacitive performance result from the nature of wide‐bandgap Co_3_O_4_ semiconductor.^15^, ^16^ To boost the electrochemical performance of Co_3_O_4_, the synthesis of composites with highly conductive carbon materials (carbon fiber, carbon nanotubes, graphene, etc.) has usually been adopted.^17^, ^18^, ^19^ However, the high specific capacitance of Co_3_O_4_ cannot be efficiently released due to the sluggish surface chemical reactivity.

Tailoring electroactive materials into diverse functional architectures has triggered unprecedented innovation in the promotion of energy storage devices. Nanostructured electrodes with higher specific surface areas and shorter diffusion paths can achieve better electrochemical performance than that of traditional bulk materials.^20^, ^21^ Among various structures, large works have been carried out to synthesize hollow nanostructures in view of their many advantages such as high surface‐to‐volume ratio, enlarged electrode/electrolyte interface, low density, short mass‐ and charge‐transport lengths, and high volumetric loading capacity.^22^ Many strategies have been utilized to synthesize the hollow TMO nanostructures including hollow nanoparticles (HNPs), hollow nanospheres, hollow nanocubes, hollow nanowires, and hollow hierarchical structures, for SCs with remarkable electrochemical performance.^23^, ^24^, ^25^ However, these methods suffer from relatively complex experimental conditions, low space‐time yields, and high technical requirements, and thus are not effective for large‐scale synthesis. In addition, these strategies mostly engineer shell‐like hollow structures with limited specific surface area. It is well known that the electrochemical performance of electrode materials is originally dominated by the surface or near‐surface chemistry, where the highly specific surface can afford abundant electroactive sites for the fast and efficient redox reactions.^26^ Unfortunately, the traditional shell‐like structure can only provide limited‐access reactive sites for participating in electrochemical reactions.^27^, ^28^ Therefore, despite progress, the scalable synthesis of hollow nanostructure with more accessible surface area still remains challenging because of the limitations of synthesis strategies.

Fundamentally, monodisperse NPs provide a model system for better understanding the structure–property relationship of nanostructured electrodes, because their size, shape, and surface chemistry can be precisely controlled.^29^ Herein, self‐assembly of polydopamine (PDA) was used to boost a simple and scalable fabrication for the bubble‐nanofiber‐structured and bubble‐nanosheet‐structured Co_3_O_4_ supraparticle composites. Compared to solid bulk materials and traditionally hollow structured NPs, hierarchical structural supraparticles have been demonstrated to be more suitable electrode materials for energy storage.^30^, ^31^ Because the hollow structure can afford more accessible reactive sites, leading to a larger energy density, the “porous shell” composed of individual ultrasmall NPs can promote the transport of the electrolyte to the active surface, thus shortening transport length for both ions and charges, resulting in a higher power density.^32^ In addition, after the carbonization of organic PDA ligands, the thin carbon layer acts as a “bridge” between these individual Co_3_O_4_ NPs, which significantly lowers interparticle resistance for electronic and ionic transport.^33^ In addition, the hierarchical structure can mitigate the damage of Co_3_O_4_ during the electrochemical reaction, leading to a better electrochemical stability.^34^ However, to the best of our knowledge, there are very few reports on the capacitive application of supraparticle structural electrodes. As we expected, synthesizing hollow supraparticles by connecting with highly conductive materials has proved to be an efficient strategy for significantly improving the energy storage performance of Co_3_O_4_ NPs. Specifically, the as‐prepared reduced graphene oxide (RGO)/hollow supraparticle (HSP)‐Co_3_O_4_ (bubble‐nanosheet‐structured Co_3_O_4_ supraparticle) electrode delivers a high specific capacitance of 1435 F g^−1^ at a current density of 1 A g^−1^ and retain 833 F g^−1^ even at 60 A g^−1^ as well as high electrochemical stability, which is higher than most reported Co_3_O_4_‐based electrodes. Furthermore, the RGO/HSP‐Co_3_O_4_‐based asymmetric supercapacitor (ASC) device can yield a maximum energy density of 51 W h kg^−1^ and remarkable cycling stability (92.3% retention after 10 000 cycles).

## 2 Results and Discussion

**Figure 1:**
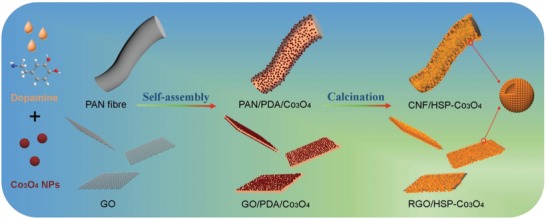
Schematic illustration of the fabrication of bubble‐nanofiber‐structured and bubble‐nanosheet‐structured Co_3_O_4_ supraparticles composite materials.

The bubble‐structured Co_3_O_4_ supraparticles anchored on carbon nanofiber (CNF; bubble nanofiber structure, CNF/HSP‐Co_3_O_4_) and reduced graphene oxide (RGO; bubble nanosheet structure, RGO/HSP‐Co_3_O_4_) were facilely fabricated via a solution self‐assembly strategy as schematically illustrated in **Figure** **1**; the detailed synthesis procedures are given in the “Experimental Section” and in the Supporting Information. The carbon nanofiber and graphene oxide (GO) were first modified with the mussel‐inspired self‐polymerized PDA, which shows strong adhesion on essentially any organic and inorganic substrates.^35^ The PDA modification to facilitate the assembly of NPs, combined with its adhesive properties, provides a simple method toward synthesizing supraparticles with densely loaded functional NPs. In addition, many hydrophilic groups endow the cross‐linked PDA with a porous hydrogel‐like structure, which facilitates free access for ultrasmall NPs.^36^ Most importantly, the PDA coating bridges the NPs through weak noncovalent interactions, minimizing passivation of the NPs. Furthermore, the PDA can be converted into a thin and conformal carbon layer by an in situ annealing process. The thin carbon layer not only shortens the interparticle spacing for fast ion and electron transport between individual NPs but also effectively prevents NPs aggregation during long‐term cycling.

**Figure 2:**
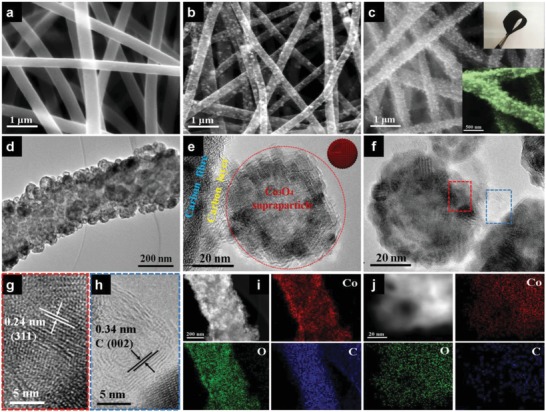
SEM images of a) pure CNF and b) CNF/H‐Co_3_O_4_. c) SEM image of bubble‐nanofiber‐structured CNF/HSP‐Co_3_O_4_ and the digital image of the prepared freestanding and flexible film. d) TEM image of a single fiber of CNF/HSP‐Co_3_O_4_. e) HRTEM image of the region between CNF and Co_3_O_4_ supraparticle. Inset: schematic illustration of supraparticle. f) HRTEM image of the region between two Co_3_O_4_ supraparticles. g,h) HRTEM images of the red and blue regions marked in panel (f), respectively. i,j) EDS element mappings of CNF/HSP‐Co_3_O_4_ and a single hollow Co_3_O_4_ supraparticle, respectively.

The scanning electron microscopy (SEM) images of CNF (derived from the carbonation of polyacrylonitrile (PAN) fiber), CNF/hollow Co_3_O_4_ NPs (CNF/H‐Co_3_O_4_, synthesized in the same procedure with CNF/HSP‐Co_3_O_4_ except PDA modification), and CNF/HSP‐Co_3_O_4_ display the evolution of surface morphology of the samples at different fabrication steps. The average diameter of pure CNF is ≈250 nm with a smooth surface and uniform distribution (**Figure** **2**a). The surface roughness of PAN fiber lightly increases after PDA coating (Figure S1, Supporting Information). For CNF/H‐Co_3_O_4_, the Co_3_O_4_ nanoparticles gather on the surface of CNF with low mass loading and disordered distribution in the absence of PDA (Figure 2b). Obviously, after being modified with PDA, a large number of bubble‐like Co_3_O_4_ were uniformly coating the surface of CNF, resulting in thicker fibrous CNF/HSP‐Co_3_O_4_ with a diameter of ≈400 nm (Figure 2c). It is favorable to its self‐supported characteristics, and results in the good flexibility and freestanding capability of the CNF/HSP‐Co_3_O_4_ hybrid fiber film (inset of Figure 2c). The bubble‐nanofiber‐structured hybrid fiber can be uniformly prepared on a large scale, and the size of “bubble” can be precisely controlled (Figure S2, Supporting Information).

The bubble‐like structure was further confirmed by transmission electron microscopy (TEM) observation of the CNF decorated with well‐defined hollow structures (Figure 2d). The higher‐magnification TEM image reveals that the bubble‐like Co_3_O_4_ with a diameter of ≈50 nm was anchored on the surface of CNF by a thin carbon layer (Figure 2e). The single bubble‐like Co_3_O_4_ with a close‐packed configuration consisted of many individual Co_3_O_4_ nanoparticles (the individual Co_3_O_4_ nanoparticle with a diameter of ≈5 nm (Figure S3, Supporting Information), which formed a superstructure (i.e., supraparticle) as the schematic illustration shows (inset of Figure 2e)). The thin carbon layer bridges the bubble‐like Co_3_O_4_ supraparticle and the CNF, which act as a “net” for griping individual bubble‐like Co_3_O_4_ supraparticles. To further confirm this, the individual ultrasmall Co_3_O_4_ NPs (with a diameter of ≈5 nm) on PDA‐modified CNF were prepared, and undoubtedly, a very thin carbon layer coated an individual ultrasmall Co_3_O_4_ NP (Figure S4, Supporting Information). In addition, the neighboring bubble‐like Co_3_O_4_ supraparticles were bridged with each other via a thin carbon layer (Figure 2f). The high‐resolution TEM (HRTEM) image (Figure 2g) shows clear lattice fringes of 0.24 nm, which correspond to the (311) lattice plane of Co_3_O_4_, as confirmed by the selected area electron diffraction (SAED) pattern (Figure S5, Supporting Information). The HRTEM image (Figure 2h) further disclosed the graphitic carbon layer between neighboring bubble‐like Co_3_O_4_ supraparticles. For comparison, the TEM images of CNF/H‐Co_3_O_4_ reveal that the small hollow Co_3_O_4_ NPs with a diameter of ≈20 nm are not uniformly distributed on the CNF without PDA modification (Figure S6, Supporting Information). The formation of hollow structure can be attributed to the Kirkendall effect of transformation from Co solid NPs into Co_3_O_4_ hollow NPs during the annealing process.^37^ Phase transformation of Co into Co_3_O_4_ was identified by X‐ray diffraction (XRD) analysis (Figure S7, Supporting Information). In addition, high‐angle annular dark‐field scanning TEM (HAADF–STEM) images and energy dispersive spectroscopy (EDS) elemental mapping images show that the Co, O, and C elements were distributed homogeneously through the entire CNF/HSP‐Co_3_O_4_ and a single hollow Co_3_O_4_ supraparticle (Figure 2i,j).

The surface chemical composition and oxidation state of CNF/HSP‐Co_3_O_4_ were investigated by X‐ray photoelectron spectroscopy (XPS), which demonstrates the presence of Co, O, C, and N in the composite (Figure S8, Supporting Information). The high‐resolution XPS spectra of Co 2p show two major peaks at 781.4 and 797.2 eV, which correspond to the Co 2p_3/2_ and Co 2p_1/2_ spin–orbit peaks, respectively; while a pair of shake‐up satellite peaks at 786.2 and 803.2 eV are characteristic of Co_3_O_4_ phase.^38^ Besides, two pairs of fitting peaks are assigned to Co^3+^ (780.7 and 796.3 eV) and Co^2+^ (782.7 and 798.1 eV), respectively. The O 1s spectra can be deconvoluted into three peaks at 530.1, 531.9, and 532.6 eV, revealing the existence of Co—O, C=O, and C—OH/C—O—C, respectively. In the C 1s high‐resolution XPS spectra, the binding energies at 284.5, 285.3, 286.4, and 287.2 eV are attributed to C—C, C—N, C=C, and C=O, respectively. The nitrogen adsorption–desorption measurements of Co_3_O_4_, CNF/H‐Co_3_O_4_, and CNF/HSP‐Co_3_O_4_ composites were conducted to obtain the specific surface area (Figure S9, Supporting Information). The CNF/HSP‐Co_3_O_4_ composites have a large specific surface area of 420 m^2^ g^−1^, which is much higher than those of Co_3_O_4_ (32 m^2^ g^−1^) and CNF/H‐Co_3_O_4_ (335 m^2^ g^−1^). The large specific surface area allows easy diffusion of electrolytes to active sites and facilitates fast transportation of electrolyte ions, thus greatly improving capacity and rate capability.

**Figure 3:**
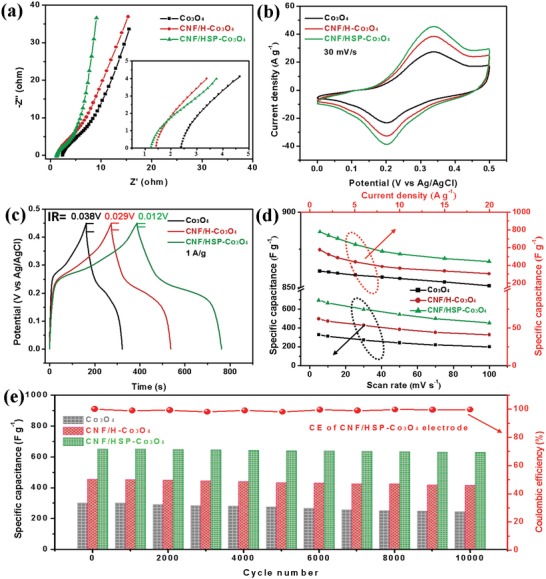
Comparison of electrochemical performance of Co_3_O_4_, CNF/H‐Co_3_O_4_, and CNF/HSP‐Co_3_O_4_ in a three‐electrode configuration. a) Nyquist plots. b) CV curves at a scan rate of 30 mV s^−1^. c) GCD curves at a current density of 1 A g^−1^. d) The specific capacitances calculated from CV and GCD curves. e) Cycling performance of Co_3_O_4_, CNF/H‐Co_3_O_4_, and CNF/HSP‐Co_3_O_4_ electrodes at a current density of 5 A g^−1^, respectively, and the corresponding Coulombic efficiency of CNF/HSP‐Co_3_O_4_ electrode.

The electrochemical performance of these electrodes was first measured in a three‐electrode cell in a voltage window of 0–0.5 V (vs Ag/AgCl). Electrochemical impedance spectroscopy (EIS) was used to investigate the electron/ion transport behavior of the electrodes. The obtained Nyquist plots (**Figure** **3**a) show that the bulk series resistance (*R*_s_) of the Co_3_O_4_ electrode has been largely reduced by using CNF as conductive support. Because of the carbon thin layer derived from PDA, the CNF/HSP‐Co_3_O_4_ electrode exhibits a smaller *R*_s_ (≈1.2 Ω) and a more ideal straight line in the low‐frequency region compared with its counterparts (Co_3_O_4_ and CNF/H‐Co_3_O_4_), which benefited from favorable reaction kinetics and lower internal resistance. Comparison of the cyclic voltammetry (CV) curves of Co_3_O_4_, CNF/H‐Co_3_O_4_, and CNF/HSP‐Co_3_O_4_ composite electrodes at a scan rate of 30 mV s^−1^ show substantially larger capacitance of CNF/HSP‐Co_3_O_4_ than Co_3_O_4_ and CNF/H‐Co_3_O_4_ electrodes (Figure 3b). These results reveal that the participation of CNF can improve the capacitor performance of Co_3_O_4_ and that the presence of PDA can not only increase the mass loading of Co_3_O_4_, but it can also convert into carbon layer to bridge the CNF and Co_3_O_4_ as a “highway” for electrochemical reactions. The galvanostatic charge–discharge (GCD) curves of the three electrodes reveal that the CNF/HSP‐Co_3_O_4_ electrode holds the longest discharge time and smallest voltage drop in the discharge process, displaying the highest specific capacitance and better electrical and ionic conductivities than its counterparts (Figure 3c). Obviously, the CNF/HSP‐Co_3_O_4_ electrode delivered the highest specific capacitance of 694.4 F g^−1^ at 5 mV s^−1^ (Figure 3d), whereas only 495.6 and 327.4 F g^−1^ were obtained for CNF/H‐Co_3_O_4_ and Co_3_O_4_ electrodes, respectively. In addition, the high capacity retention of 65.3% obtained even at a high scan rate of 100 mV s^−1^, displaying that the bubble‐like CNF/HSP‐Co_3_O_4_ electrode has a superior rate capability. Moreover, this CNF/HSP‐Co_3_O_4_ electrode also had long‐term electrochemical stability, which retained ≈96.9% of its initial capacitance after 10 000 cycles with the entire processes exhibiting nearly 100% Coulombic efficiency, which is higher than its counterparts (Figure 3e).

The remarkable electrochemical performance of bubble‐nanofiber‐structured CNF/HSP‐Co_3_O_4_ electrode can be mainly attributed to its synergic features of high‐conductivity net‐like CNF film, hierarchical structural bubble‐like Co_3_O_4_ supraparticles, and the thin carbon “bridge.” First, the electrospun CNF has 3D interconnected hierarchical porous structures, which act as a strong reservoir for ions, guarantee efficient contact between Co_3_O_4_ supraparticles and electrolytes even at high rates, and greatly reinforce the diffusion kinetics within electrode. Specifically, the hollow structure of the bubble‐like Co_3_O_4_ supraparticles anchored on the surface of CNF to form high‐surface‐area morphology for fast charge transport, and the supraparticle structure assembled by individual ultrasmall Co_3_O_4_ NPs provides numerous electroactive sites for efficient electrochemical reactions. Furthermore, the bubble‐like Co_3_O_4_ supraparticles bridged with each other and the highly conductive CNF via a thin carbon layer, thus strongly enhancing the transportation of electronic and ion in whole electrode. This maximized the utilization rate of electroactive sites of individual Co_3_O_4_ NPs for better electrochemical performance.

**Figure 4:**
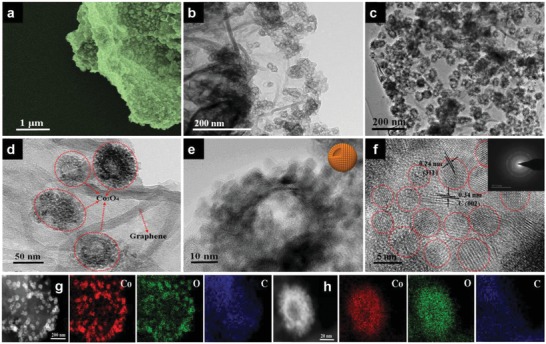
a) SEM image of bubble‐nanosheet‐structured RGO/HSP‐Co_3_O_4_. b) TEM image of RGO/H‐Co_3_O_4_. c,d) TEM images of RGO/HSP‐Co_3_O_4_. e) HRTEM image of a single hollow Co_3_O_4_ supraparticle. f) HRTEM image of Co_3_O_4_ nanoparticles, and the inset shows the corresponding SAED pattern. g,h) HAADF–STEM images and corresponding EDS element images of RGO/HSP‐Co_3_O_4_ and a single hollow Co_3_O_4_ supraparticle, respectively.

The SEM image of RGO/HSP‐Co_3_O_4_ indicates that the bubble‐like Co_3_O_4_ supraparticles are uniformly and densely distributed on the surface of RGO sheet (**Figure** **4**a). The low mass loading and small size of Co_3_O_4_ hollow NPs adhered to the surface of RGO sheet for the RGO/H‐Co_3_O_4_ sample synthesized without PDA modification (Figure 4b). After being modified with PDA, the bubble‐nanosheet‐structured RGO/HSP‐Co_3_O_4,_ with more quantity and density of bubble‐like Co_3_O_4_ supraparticles, was uniformly anchored to the RGO sheet than RGO/H‐Co_3_O_4_ (Figure 4c). The bubble‐nanosheet‐structured graphene composite can be controllably prepared with different “bubble” sizes (Figure S10, Supporting Information). The HRTEM image reveals that a single bubble‐like Co_3_O_4_ supraparticle was composed of many ultrasmall Co_3_O_4_ NPs (Figure 4d,e). The HRTEM image reveals the (311) facet of Co_3_O_4_ and (002) facet of graphitic carbon (Figure 4f). The SAED pattern also reveals the highly crystalline structure of Co_3_O_4_. These results were further confirmed by XRD data (Figure S11, Supporting Information). The HAADF–STEM images and corresponding elemental mapping images show that Co, O, and C are homogeneously distributed throughout RGO/HSP‐Co_3_O_4_ and on a single hollow Co_3_O_4_ supraparticle (Figure 4g,h). The surface chemical composition and oxidation state of RGO/HSP‐Co_3_O_4_ were further investigated by XPS, which demonstrates the presence of Co, O, and C in the composite (Figure S12, Supporting Information). In the high‐resolution Co 2p XPS spectra, the binding energies at around 780.9 and 796.7 eV are associated with Co 2p_3/2_ and Co 2p_1/2_, respectively. The peaks at 780.4 and 796.3 eV can be ascribed to Co^3+^, whereas the peaks at 782.6 and 798.3 eV correspond to Co^2+^. In the high‐resolution O 1s XPS spectra, the binding energies located at 530.4, 532.0, and 533.3 eV are attributed to the presence of Co_3_O_4_ lattice oxygen, surface‐adsorbed —OH functional groups, and residual oxygen‐containing groups in RGO, respectively. In the high‐resolution C 1s XPS spectra, the binding energies at 284.7, 285.2, 286.4, and 287.3 eV are attributed to C—C, C—N, C=C, and C=O, respectively. The dominated C—C bond in the C 1s spectra signifies the reduction of GO to RGO. The nitrogen adsorption–desorption measurements reveal that the RGO/HSP‐Co_3_O_4_ exhibits a larger specific surface area of 664 m^2^ g^−1^ than RGO/H‐Co_3_O_4_ (465 m^2^ g^−1^) (Figure S13, Supporting Information).

**Figure 5:**
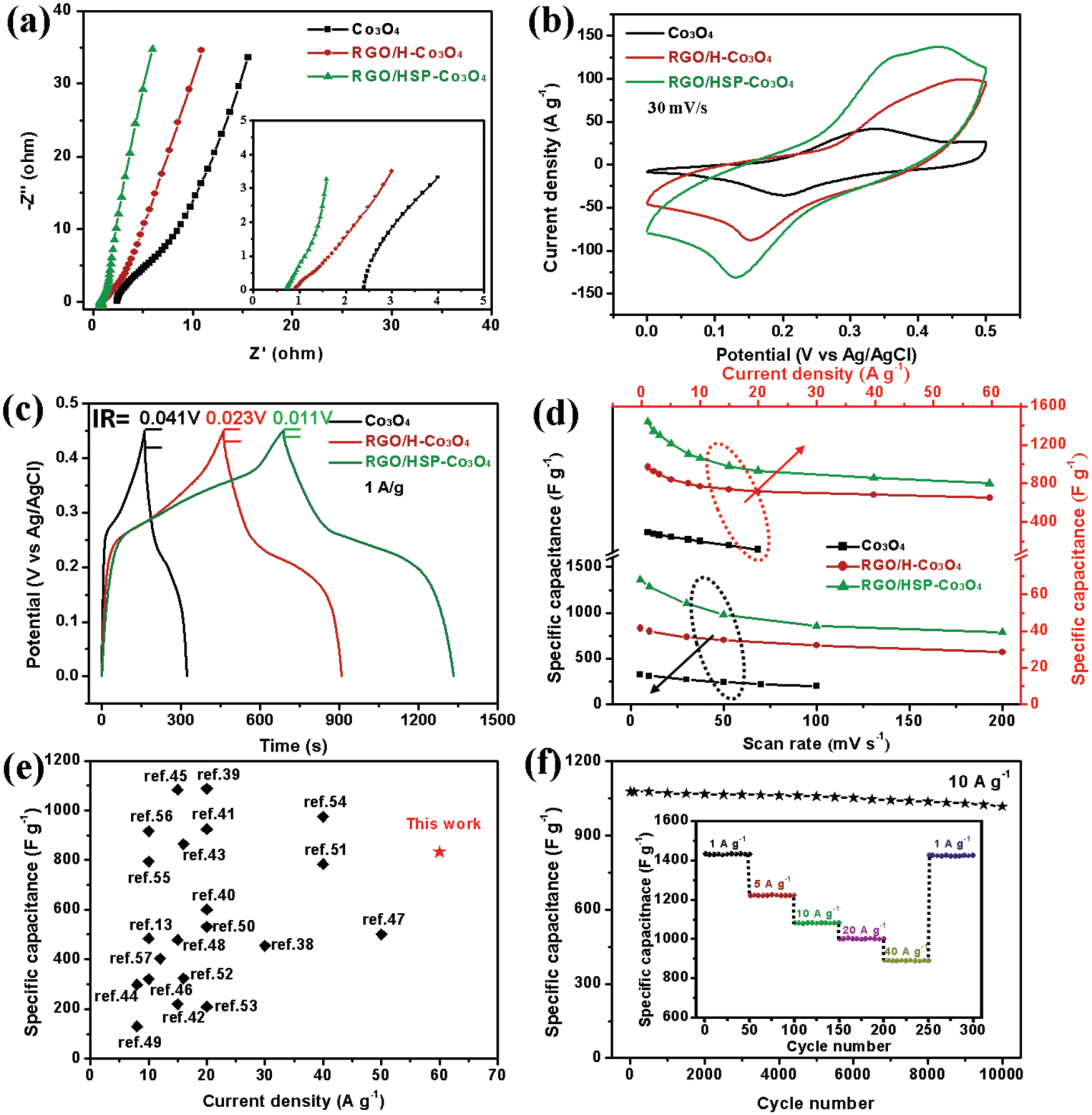
Comparison of electrochemical performance of Co_3_O_4_, RGO/H‐Co_3_O_4_, and RGO/HSP‐Co_3_O_4_ in a three‐electrode configuration. a) Nyquist plots. b) CV curves at a scan rate of 30 mV s^−1^. c) GCD curves at a current density of 1 A g^−1^. d) The specific capacitances calculated from CV and GCD curves. e) Comparison of the specific capacitances of RGO/HSP‐Co_3_O_4_ and other Co_3_O_4_‐based electrodes in the literature. All the specific capacitance values correspond to the highest current density reported in the literature. f) Long‐term cycling performance of the RGO/HSP‐Co_3_O_4_ electrode at a current density of 10 A g^−1^ for 10 000 cycles and cycling stability of the RGO/HSP‐Co_3_O_4_ electrode at consecutively various current densities.

To investigate the superior electrochemical performance of the bubble‐nanosheet‐structured RGO/HSP‐Co_3_O_4_ electrode, two counterparts (Co_3_O_4_ and RGO/H‐Co_3_O_4_) were also measured for comparison. The EIS curves (**Figure** **5**a) of Co_3_O_4_, RGO/H‐Co_3_O_4_, and RGO/HSP‐Co_3_O_4_ reveal that the RGO/HSP‐Co_3_O_4_ electrode has the lowest *R*_s_ (≈0.76 Ω). From CV curves in Figure 5b, the pair of redox peaks can be seen in all of electrodes, which result from the Faradaic reactions of cobalt oxide in KOH solution. The RGO/HSP‐Co_3_O_4_ shows the largest CV curve area and redox peak intensity compared to Co_3_O_4_ and RGO/H‐Co_3_O_4_ electrodes, implying a significantly improved specific capacitance and faster redox reaction kinetics processes. The GCD curves (Figure 5c) of the three samples show that the discharging time of RGO/HSP‐Co_3_O_4_ is much longer and has smaller internal resistance (IR) drop than other samples, indicating higher specific capacitance and faster reaction kinetics. Figure 5d reveals that the RGO/HSP‐Co_3_O_4_ electrode has the highest specific capacitance of 1360 F g^−1^ at 5 mV s^−1^, which was only 830 and 327 F g^−1^ for RGO/H‐Co_3_O_4_ and Co_3_O_4_ electrodes, respectively. It is worth noting that 57.8% of capacitance was maintained even at a high scan rate of 200 mV s^−1^, indicating that the bubble‐nanosheet‐structured electrode shows excellent rate performance. Figure 5e compares the specific capacitance of the RGO/HSP‐Co_3_O_4_ electrode and other Co_3_O_4_‐based materials measured at high current densities. Obviously, the RGO/HSP‐Co_3_O_4_ electrode can effectively work at higher current density, while the specific capacitance is higher than most values in the literature.^13^, ^38^, ^39^, ^40^, ^41^, ^42^, ^43^, ^44^, ^45^, ^46^, ^47^, ^48^, ^49^, ^50^, ^51^, ^52^, ^53^, ^54^, ^55^, ^56^, ^57^ The high rate performance can be attributed to the bubble nanosheet structure and fast electrolyte diffusion inside the whole electrode. It has been proved that the rate performance of electrode materials significantly depends on the electrolyte diffusion rate, both inside the bulk phase and in the whole porous electrode. Thus, the hierarchical bubble‐nanosheet‐structured Co_3_O_4_ supraparticle composites are desirable. The cycling stability of RGO/HSP‐Co_3_O_4_ electrode was measured at 1, 5, 10, 20, and 40 A g^−1^ for 50 cycles, respectively, and 98.7% of the initial capacity was retained when the charge/discharge rate was set back to 1 A g^−1^ (Figure 5f). In addition, a long‐term cycling test was carried out at 10 A g^−1^, and 94.3% recovery of the initial capacitance obtained after 10 000 cycles, implying the excellent electrochemical cycling stability of the bubble‐nanosheet‐structured RGO/HSP‐Co_3_O_4_ electrode. The improved capacitance and redox reaction kinetics can be attributed to the hollow structural and compositional advantages of bubble‐nanosheet‐structured RGO/HSP‐Co_3_O_4_ electrode.

**Figure 6:**
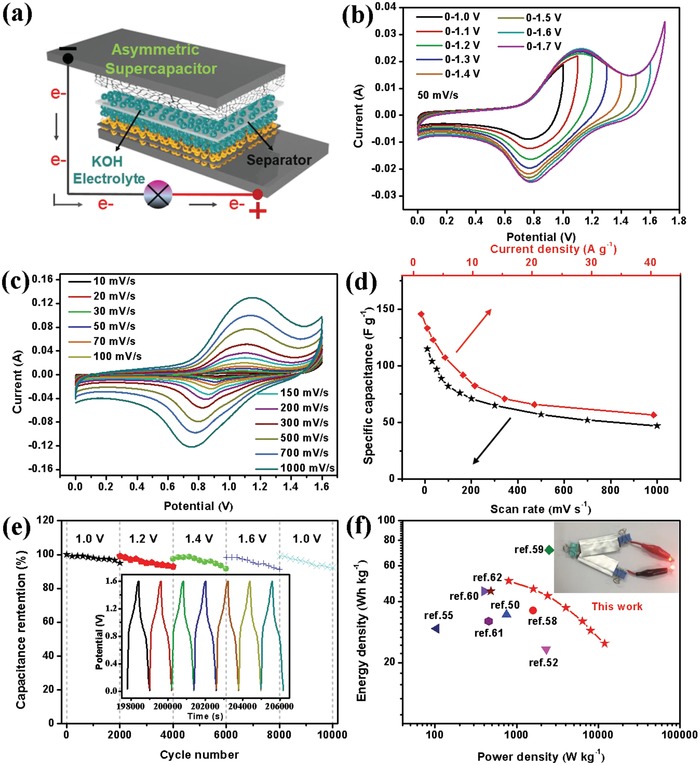
a) Schematic diagram of the fabricated ASC by RGO/HSP‐Co_3_O_4_ and graphene foam with a filter paper as separator in 2 m KOH solution. b) CV curves of the as‐assembled ASC measured at different operating voltages at a constant scan rate of 50 mV s^−1^. c) CV curves of ASC at different scan rates from 10 to 1000 mV s^−1^. d) Specific capacitance of ASC calculated from CV and GCD curves. e) Cycle performance of the ASC device measured at a current density of 5 A g^−1^ under various potential windows for 10 000 cycles. f) Ragone plots related to energy and power densities of the ASC device compared with literature results based on those of Co_3_O_4_‐based ASCs. Inset: photographic image of a red LED operating with two ASCs in series.

To evaluate the possibility of as‐synthesized bubble‐nanosheet‐structured RGO/HSP‐Co_3_O_4_ electrode's practical applications, aqueous ASC was fabricated with the RGO/HSP‐Co_3_O_4_ electrode as cathode and the graphene foam electrode as anode, respectively, in a 2 m KOH electrolyte (**Figure** **6**a). The porous graphene foam was fabricated by the chemical vapor deposition (CVD) method (Figure S14, Supporting Information). The capacitive performance of the porous graphene foam was measured and exhibited superior electrical double‐layer capacitive characteristic as for rectangular CV curves, symmetric GCD curves, and high electrochemical stability (Figure S15, Supporting Information). Before fabricating ASC device, the mass loading of both cathode and anode was balanced according the equation given in the Supporting Information. The CV curves under their separated potential windows were recorded at a scan rate 30 mV s^−1^ (Figure S16a, Supporting Information). The low *R*_s_ value of 0.42 Ω for ASC device (Figure S16b, Supporting Information) reveals the high electrical conductivity of both cathode and anode. According to the CV curves of the ASC device measured at different voltage windows (Figure 6b), the stable working voltage can be extended to 1.6 V. Surprisingly, even at a high scan rate of 1000 mV s^−1^, the identical shape of CV curves can still be retained without obvious distortion (Figure 6c), indicating the ideal capacitive behavior and excellent rate performance of the ASC device. The ASC has a high specific capacitance of 115 F g^−1^ at 10 mV s^−1^ (Figure 6d), and 47 F g^−1^ was retained even at 1000 mV s^−1^, further confirming the high capacitance and excellent rate performance. The cycle life of the ASC was tested through a cyclic charge/discharge process at 5 A g^−1^ at different working potential windows for 10 000 cycles (Figure 6e). The capacitance retention showed obvious fluctuation with the changed voltage window, but the ASC device still maintained 92.3% retention of its initial capacitance after 10 000 cycles. Based on the mass loading of the active material on both cathode and anode, the ASC exhibited a maximum energy density of 51 W h kg^−1^ at a power density of 800 W kg^−1^ (Figure 6f), which is comparable to, or even higher than, those of state‐of‐the‐art Co_3_O_4_‐based ASCs.^50^, ^52^, ^55^, ^58^, ^59^, ^60^, ^61^, ^62^ The specific energy and power densities of our ASC device are also comparable to other electrode materials reported in literatures, such as carbon tube/NiCo_2_S_4_ nanotube//AC (27.7 W h kg^−1^ at 263.6 W kg^−1^),^63^ NiO/C‐HS//AC (30.5 W h kg^−1^ at 193 W kg^−1^),^64^ GQDs/MnO_2_‐3//NG (118 W h kg^−1^ at 12 351 W kg^−1^),^65^ Co_3_O_4_/PANI//AC (41.5 W h kg^−1^ at 800 W kg^−1^),^66^ G@NiO‐1//NGH (52.6 W h kg^−1^ at 800 W kg^−1^),^67^ NiMoO_4_//carbon nanotube film (54.3 W h kg^−1^ at 4344 W kg^−1^),^68^ CuS–AC//AC (24.88 W h kg^−1^ at 800 W kg^−1^),^69^ NiCo_2_O_4_ HNPs//AC (71 W h kg^−1^ at 1852 W kg^−1^),^70^ (Note: activated carbon (AC), carbon hollow spheres (C‐HS), graphene quantum dots (GQDs), nitrogen‐doped graphene (NG), polyaniline (PANI), nitrogen‐doped graphene hydrogel (NGH), HNPs) and some transition metal oxide‐ and nitride‐based supercapacitors.^71^, ^72^ Because the ASC device possessed a maximum working voltage of 1.6 V with excellent energy density, both charged ASCs in series could effectively operate a red light‐emitting diode (LED) (inset of Figure 6f).

## 3 Conclusion

In summary, a simple and scalable synthesis strategy has been developed for controllable fabrication of hierarchical bubble‐nanofiber‐structured and bubble‐nanosheet‐structured Co_3_O_4_ supraparticle composites. Benefiting from the unique nanostructure, the electrodes showed a high utilization rate of active material and fast ion/electron transport. Thus, the as‐fabricated RGO/HSP‐Co_3_O_4_ electrode delivered high specific capacitance (1435 F g^−1^/1360 F g^−1^ at 1 A g^−1^/5 mV s^−1^), excellent rate capability (833 F g^−1^/786 F g^−1^ at 60 A g^−1^/200 mV s^−1^), and remarkable cycling stability (94.3% retention after 10 000 cycles at 10 A g^−1^). Furthermore, the ASC device exhibited a high energy density of 51 W h kg^−1^ at the power density of 800 W kg^−1^, remarkable specific capacitance (142 F g^−1^/115 F g^−1^ at 1 A g^−1^/10 mV s^−1^), and excellent electrochemical stability (92.3% retention after 10 000 cycles). Our simple and controllable method for use of a hierarchical bubble‐like superstructure‐based electrode with excellent electrochemical properties opens the door to the synthesis of high‐performance electrode materials for next‐generation energy storage devices.

## 4 Experimental Section

*Materials' Synthesis*: PAN fiber films were produced by the electrospinning process as previously reported.^73^ GO was synthesized by a modified Hummers' method.^74^ Co_3_O_4_ NPs were prepared according to the literature procedures.^75^ The PAN fiber film and GO were first modified by PDA. Typically, the PAN fiber film and GO were immersed in a freshly prepared dopamine aqueous solution (1 mg mL^−1^ in 10 × 10^−3^ m Tris buffer, pH = 8.5) at 50 °C for 1 h, then washed with deionized (DI) water three times to remove the nonadhered PDA and dried under vacuum. The as‐synthesis PDA‐modified PAN fiber film and GO were immersed into 5 mg mL^−1^ Co_3_O_4_ NPs solution for 5 h at room temperature to produce PAN fiber/PDA/Co_3_O_4_ and GO/PDA/Co_3_O_4_, respectively. Finally, the PAN fiber/PDA/Co_3_O_4_ was annealed in air at 250 °C for 2 h with a heating rate of 5 °C min^−1^, followed by annealing in a N_2_ flow at 800 °C for 1 h with a heating rate of 2 °C min^−1^, and further annealed at 300 °C for 20 min in air to obtain the final product (denoted as CNF/HSP‐Co_3_O_4_). The typical mass loading of CNF/HSP‐Co_3_O_4_ is about 1.3 mg cm^−2^. The GO/PDA/Co_3_O_4_ was annealed in a N_2_ flow at 800 °C for 1 h with a heating rate of 2 °C min^−1^, and further annealed at 300 °C for 20 min in air to obtain RGO/HSP‐Co_3_O_4_. For comparison, CNF/H‐Co_3_O_4_ and RGO/H‐Co_3_O_4_ were also prepared with the same procedure of CNF/HSP‐Co_3_O_4_ and RGO/HSP‐Co_3_O_4_, respectively, without modification of PAN fiber and GO with PDA.

*Materials' Characterization*: The crystallographic information and phase purity of different samples were characterized by an XRD (PERSEE, XD‐3 with Cu Kα radiation) and EDS (Tecnai G2 F30 S‐TWIN). The morphology and microstructure features of the samples were investigated by field emission scanning electron microscopy (FE‐SEM; FEI, QuanTA‐200F), TEM (JEOL, JEM‐2100F), and HRTEM. A Micromeritics ASAP 2020 nitrogen adsorption apparatus was adopted to estimate the Brunauer–Emmett–Teller (BET) surface areas. The surface composition and valence states were analyzed by XPS on an ESCALAB 250Xi system with Al Kα irradiation.

*Electrochemical Measurements*: The electrochemical measurements were performed with CV, GCD, and EIS measurements using a CHI 660D electrochemical workstation (Chenhua, Shanghai) for single electrode and asymmetric supercapacitor devices, and 2 m KOH was used as the electrolyte. The graphene foam was synthesized according to Chen et al.'s work.^76^ The asymmetric supercapacitor was fabricated by taking the RGO/HSP‐Co_3_O_4_ and graphene foam as cathode (the typical mass loading of RGO/HSP‐Co_3_O_4_ is about 1.0 mg cm^−2^) and anode (the typical mass loading of graphene foam is about 2.0 mg cm^−2^), respectively, and a porous polymer membrane (Celgard 3501) as the separator.

## Conflict of Interest

The authors declare no conflict of interest.

## Supporting information

Supplementary
