# Supplementary material for: Co3O4 Supraparticle‐Based Bubble Nanofiber and Bubble Nanosheet with Remarkable Electrochemical Performance
Source: Adv Sci (Weinh). 2019 Apr 15;6(12):1900107. doi: 10.1002/advs.201900107 (PMC6662086; doi:10.1002/advs.201900107)
Supplement: Supplementary file 1 — Supplementary [file ADVS-6-1900107-s001.pdf]

Copyright WILEY-VCH Verlag GmbH & Co. KGaA, 69469 Weinheim, Germany, 2019.

ADVANCED SCIENCE
Open Access

# Supporting Information

for *Adv. Sci.,* DOI: 10.1002/advs.201900107

# $Co_3O_4$ Supraparticle-Based Bubble Nanofiber and Bubble Nanosheet with Remarkable Electrochemical Performance

*Jun Huang, Yingbo Xiao, Zhongyou Peng, Yazhou Xu, Longbin Li, Licheng Tan, Kai Yuan,* and Yiwang Chen**

Copyright WILEY-VCH Verlag GmbH & Co. KGaA, 69469 Weinheim, Germany, 2019.

# Supporting Information

## $Co_3O_4$ Supraparticle-Based Bubble Nanofibre and Bubble Nanosheet with Remarkable Electrochemical Performance

*Jun Huang, Yingbo Xiao, Zhongyou Peng, Yazhou Xu, Longbin Li, Licheng Tan, Kai Yuan,* and Yiwang Chen**

***Calculations:***

Prior to the fabrication of the asymmetric supercapacitor device, the mass loading of the cathode and anode were balanced according to the following equation:

$$\frac{m_+}{m_-} = \frac{C_{S-}\Delta V_-}{C_{S+}\Delta V_+} \quad (1)$$

where *m* is the mass, *Cs* is the specific capacitance, and $\Delta V_+$ and $\Delta V_-$ are is the voltage range for positive and negative electrodes, respectively.

In three-electrode system, For the CV curves, gravimetric-specific capacitance *C* (F $g^{-1}$) of electrode materials was calculated by integrating the discharge portion using the following equation:

$$C = \frac{\int IdV}{vmV} \quad (2)$$

where *I* is current (A), *v* is the potential scan rate (mV $s^{-1}$), *m* is mass of active material (mg), and *V* is the potential window (V).

The specific capacitance ($C_s$) can be also calculated by integrating the area under the GCD curve by the following equation:

$$C_s = \frac{I}{mdV/dt} \tag{3}$$

where $I$ (A) is the discharge current, $m$ (g) represents the mass of the active material, and the value of $dV$ (V)/$dt$ (s) indicates the slope of the discharge curve in the GCD measurement.

Two-electrode configuration (device measurements)

Gravimetric capacitance ($C_g$):

$$C_g = \frac{\int IdV}{vMV} \tag{4}$$

where $I$ (A) is the discharge current, $v$ is the potential scan rate (mV $s^{-1}$), $M$ is mass of active material in both electrodes (mg), and $V$ is the potential window (V).

Gravimetric energy density ($E_g$):

$$E_g = \frac{\int IVdt}{M} \tag{5}$$

Gravimetric power density ($P_g$):

$$P_g = \frac{E_g}{\Delta t} \tag{6}$$

where $\Delta t$ is the discharge time.

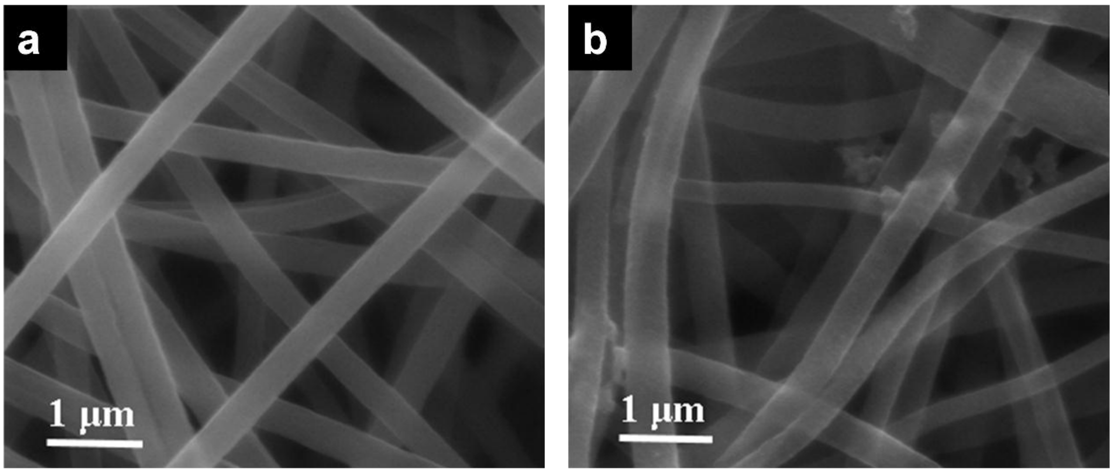


**Figure S1**. SEM images of (a) pure PAN and (b) PAN/PDA fibres.

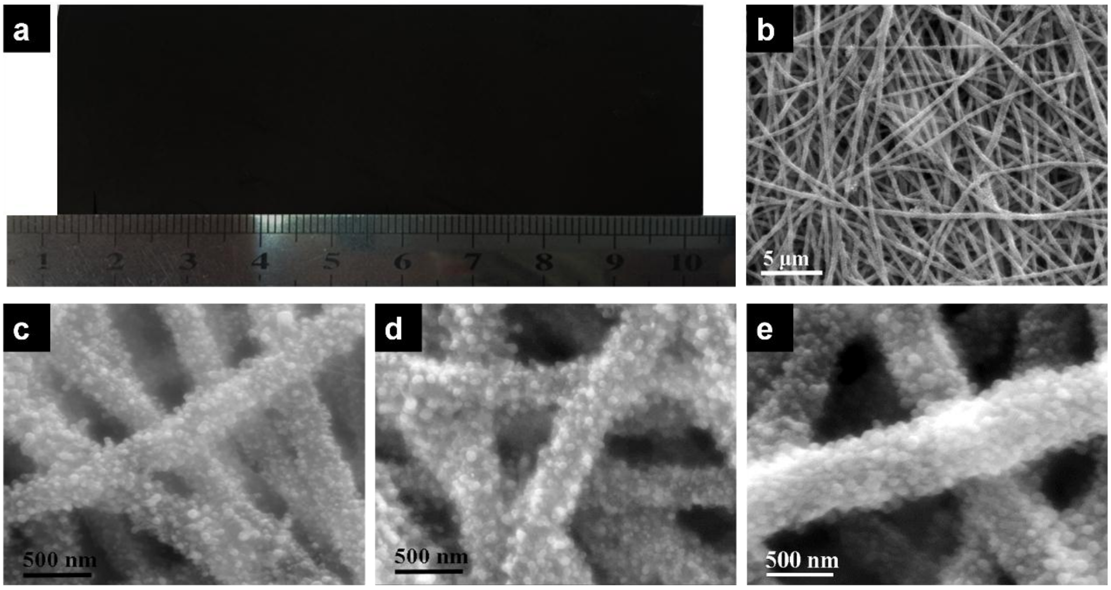


**Figure S2**. (a) The photograph of free-standing CNF/HSP-$Co_3O_4$ film (about 10 × 4 $cm^2$), (b) Low magnification SEM of CNF/HSP-$Co_3O_4$, (c, d, e) SEM images of CNF/HSP-$Co_3O_4$ fabricated with different concentrations of $Co_3O_4$ NPs.

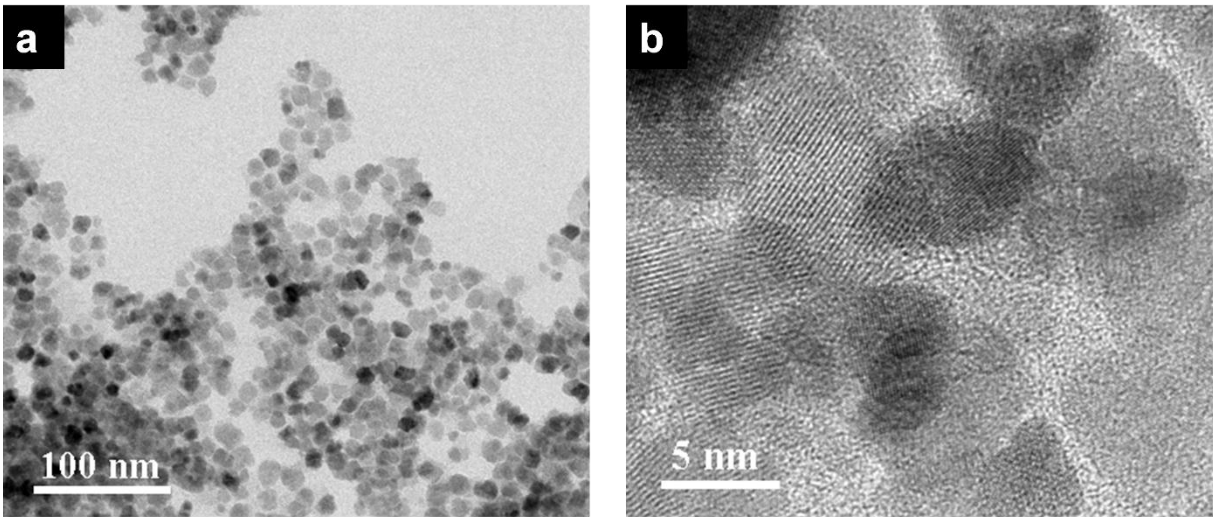


**Figure S3**. (a) TEM image and (b) HRTEM images of as-synthesis $Co_3O_4$ NPs.

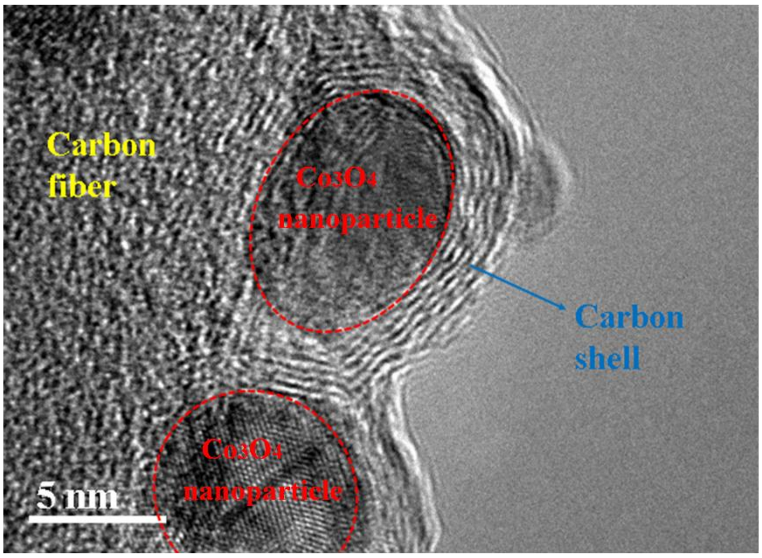


**Figure S4**. TEM image of PDA modified CNF assembly with ultra-small $Co_3O_4$ NPs, showing the presence of very thin carbon layers after in situ annealing process.

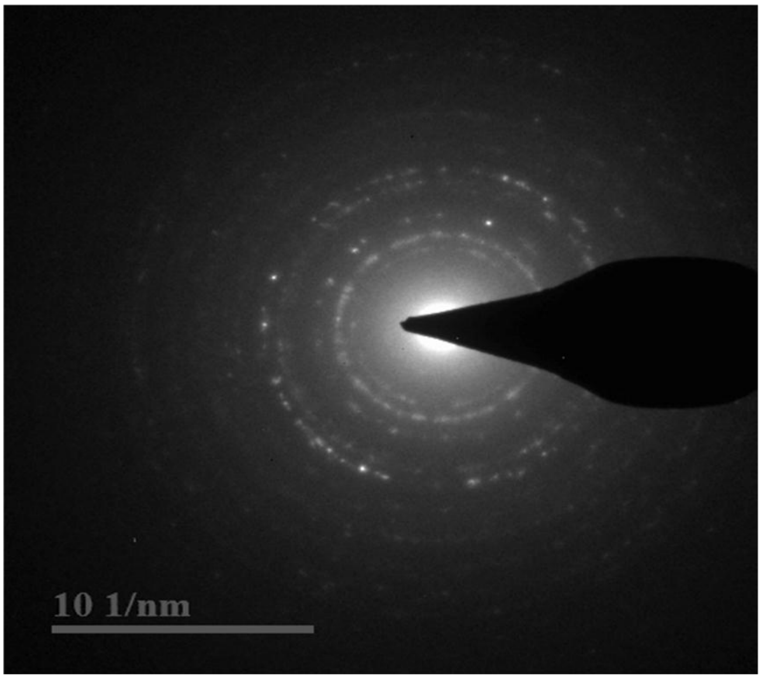


**Figure S5**. The SAED pattern of $Co_3O_4$ supraparticle.

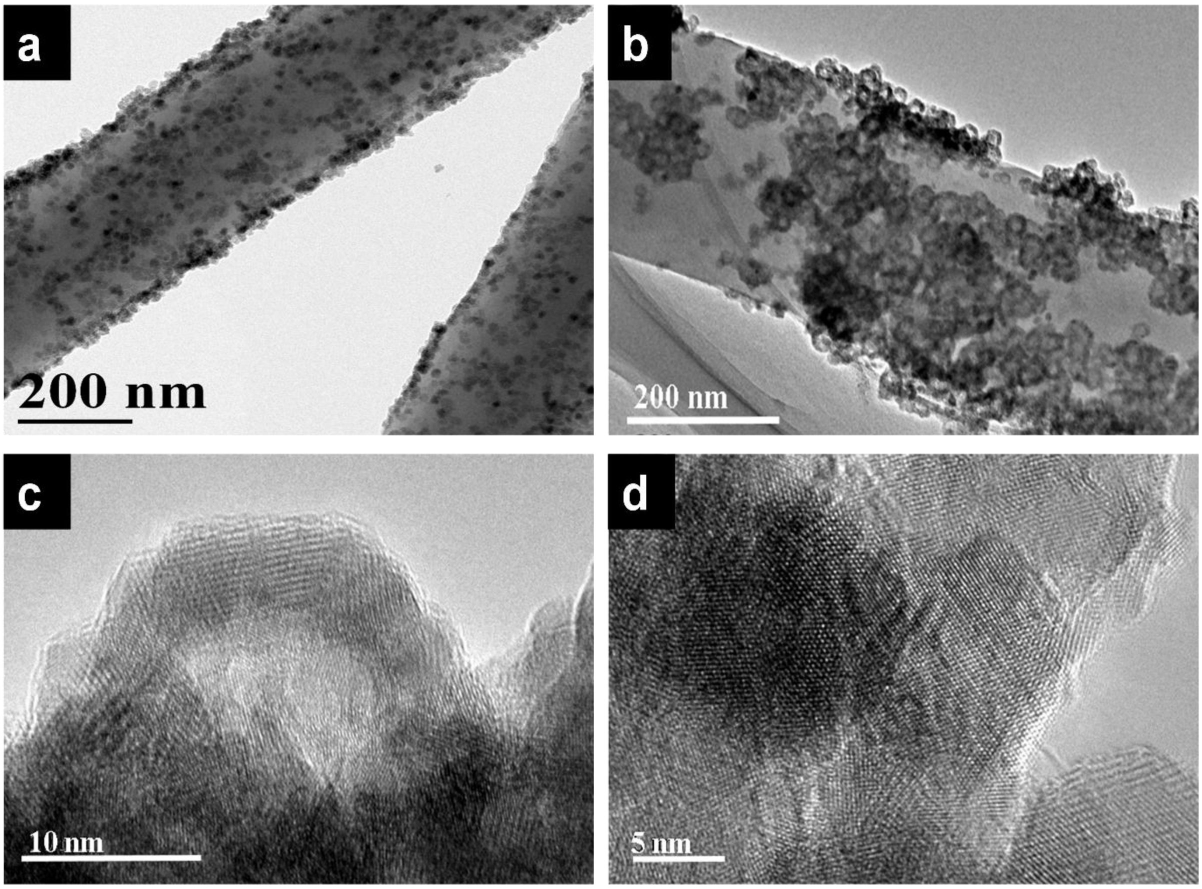


**Figure S6**. (a) TEM image of CNF/Co, (b, c, d) TEM images of CNF/H-$Co_3O_4$.

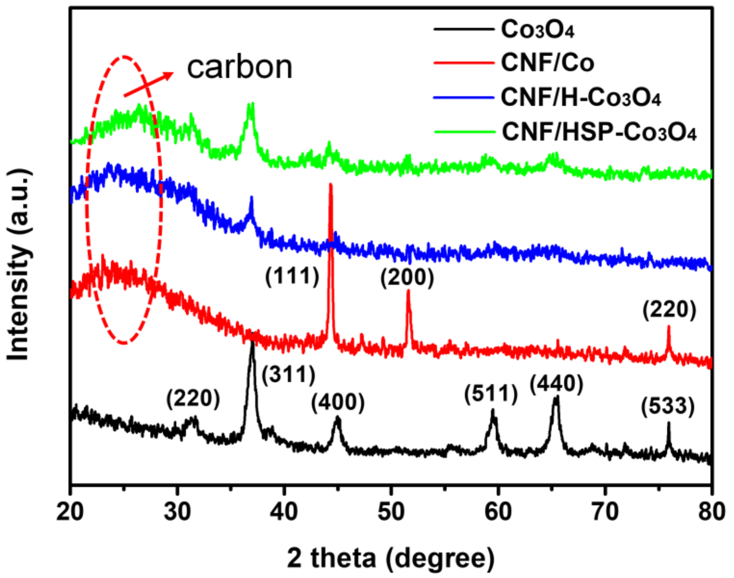


**Figure S7**. XRD patterns of $Co_3O_4$, CNF/Co, CNF/H-$Co_3O_4$, and CNF/HSP-$Co_3O_4$.

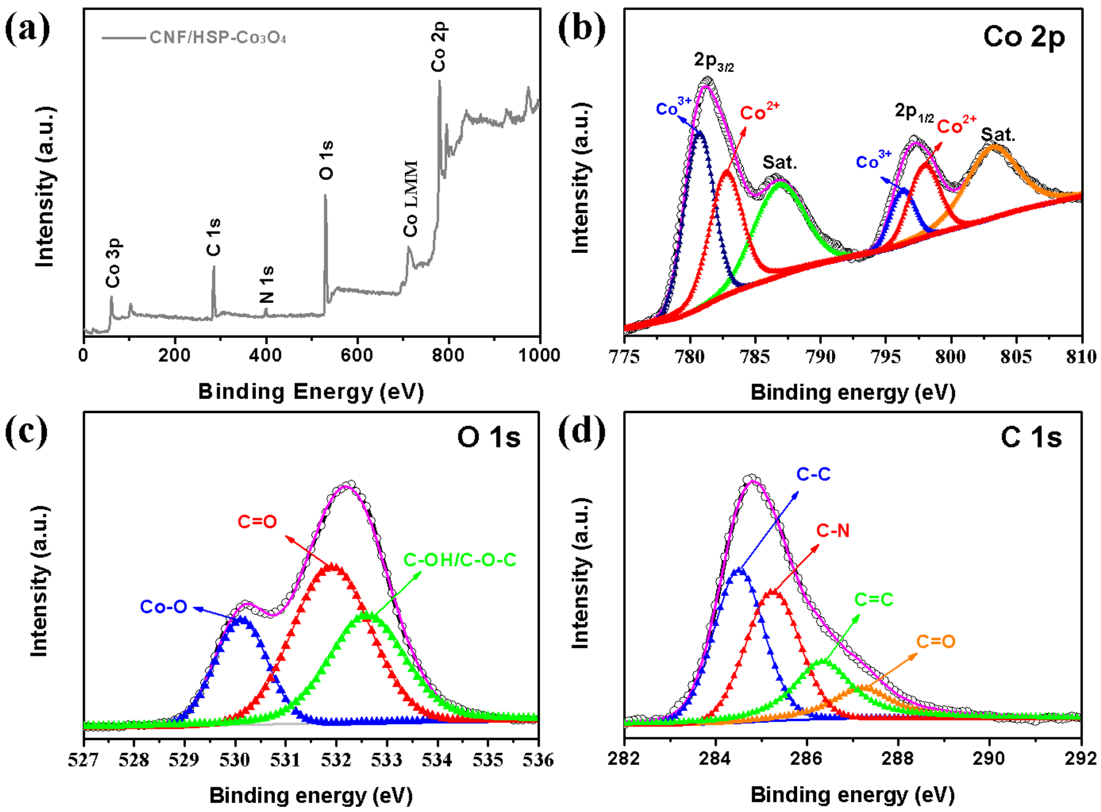


**Figure S8**. (a) XPS survey spectrum of the as-synthesized CNF/HSP-$Co_3O_4$ composite. (b) Co 2p, (c) O 1s, and (d) C 1s XPS spectra.

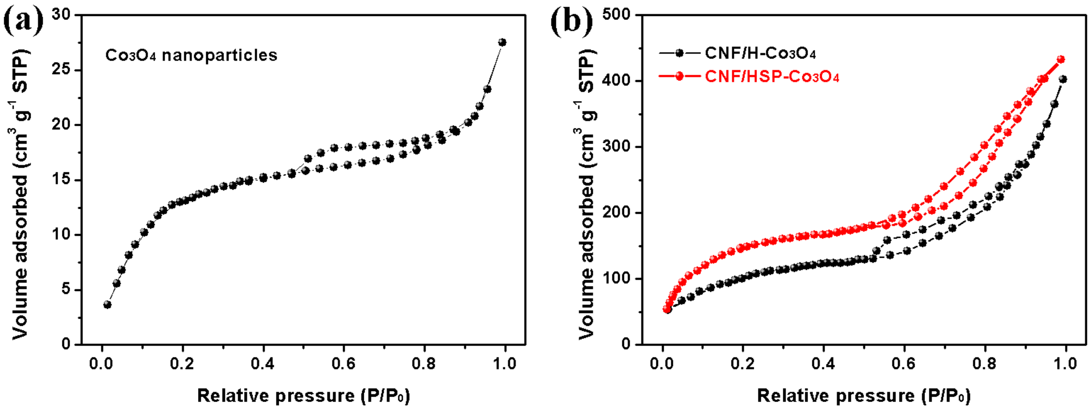


**Figure S9**. $N_2$ adsorption-desorption isotherms of (a) $Co_3O_4$ nanoparticles, (b) CNF/H-$Co_3O_4$ and CNF/HSP-$Co_3O_4$ composites.

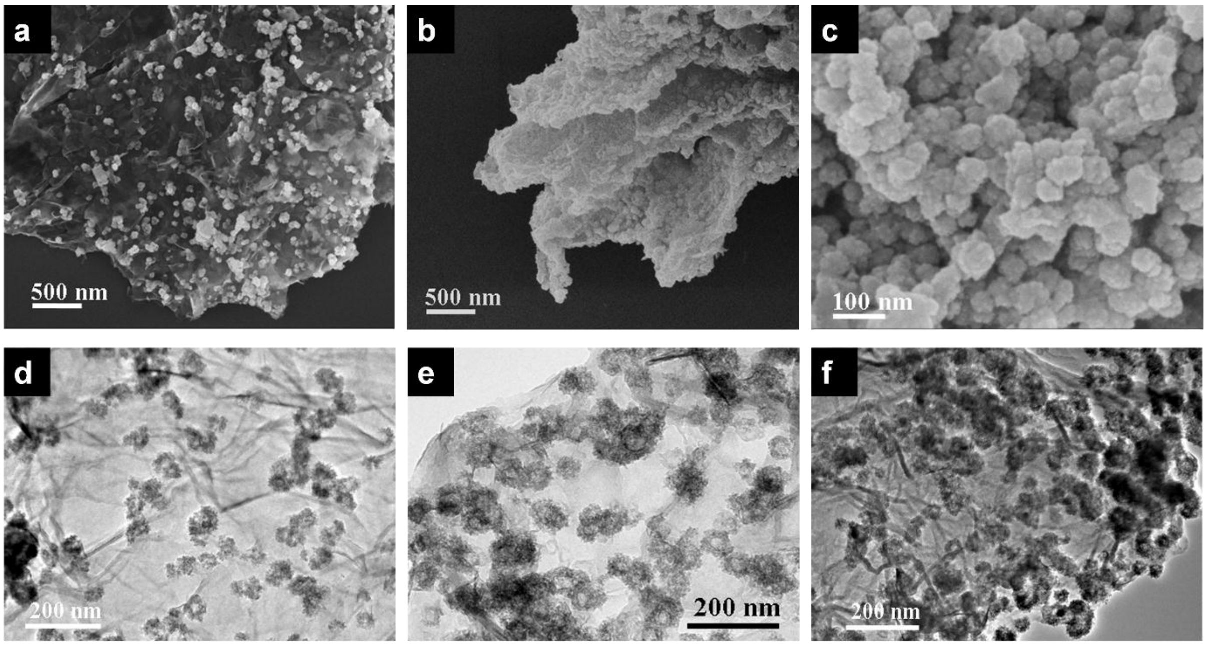


**Figure S10**. The SEM and TEM images of RGO/HSP-$Co_3O_4$ with different concentrations of $Co_3O_4$ NPs.

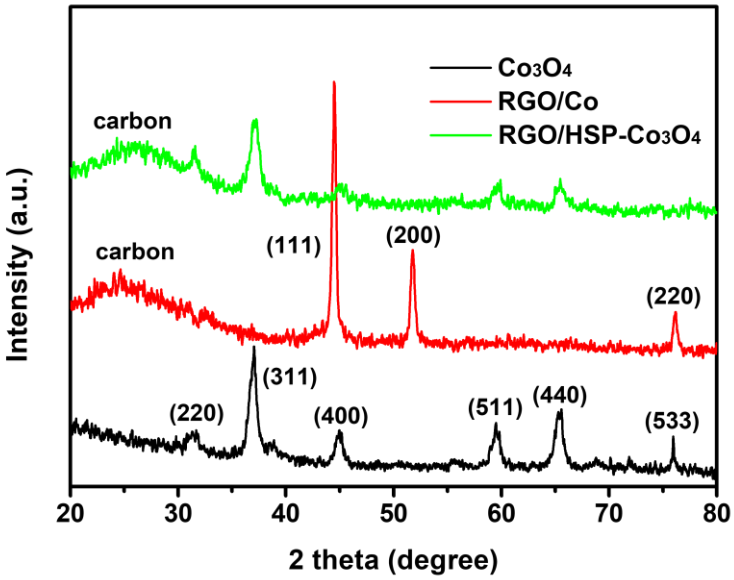


**Figure S11**. XRD patterns of $Co_3O_4$, RGO/Co, and RGO/HSP-$Co_3O_4$.

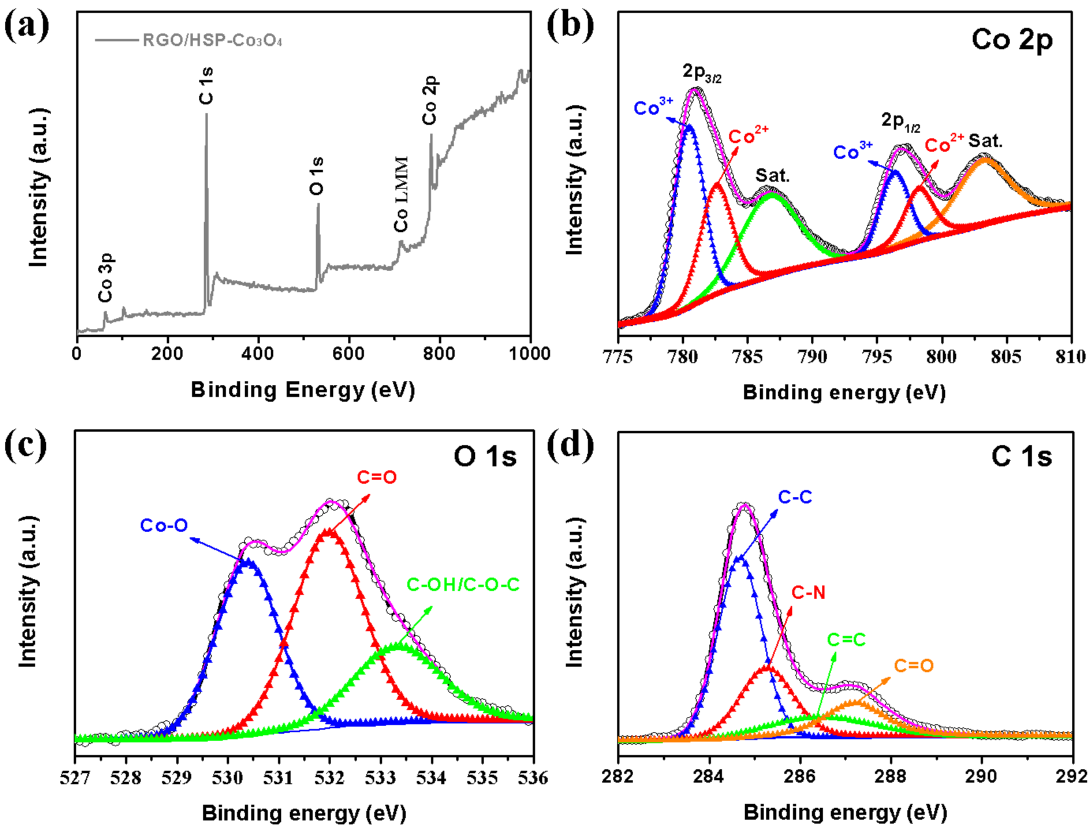


**Figure S12**. (a) XPS survey spectrum of the as-synthesized RGO/HSP-$Co_3O_4$ composite. (b) Co 2p, (c) O 1s, and (d) C 1s XPS spectra.

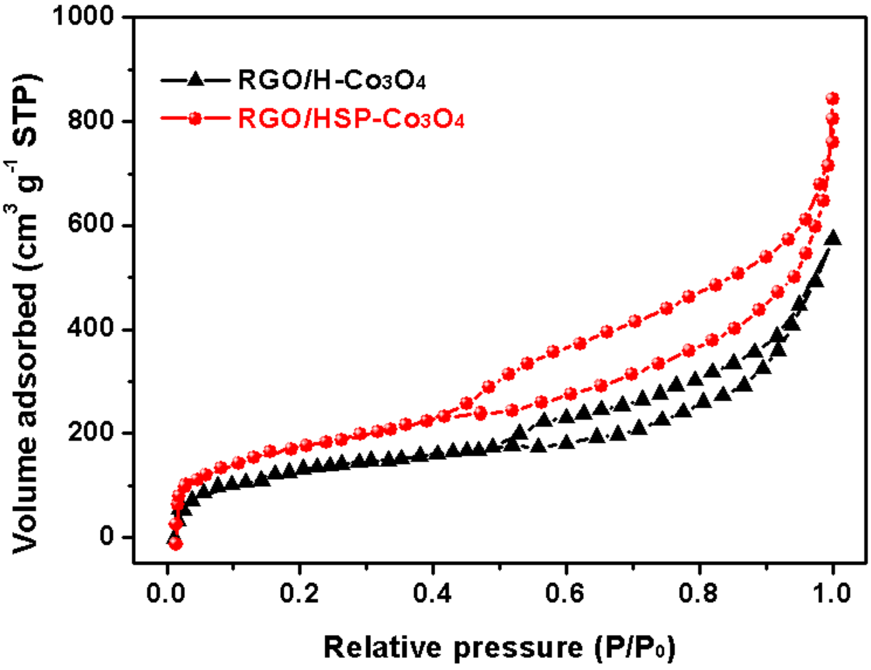


**Figure S13**. $N_2$ adsorption-desorption isotherms of RGO/H-$Co_3O_4$ and RGO/HSP-$Co_3O_4$ composites.

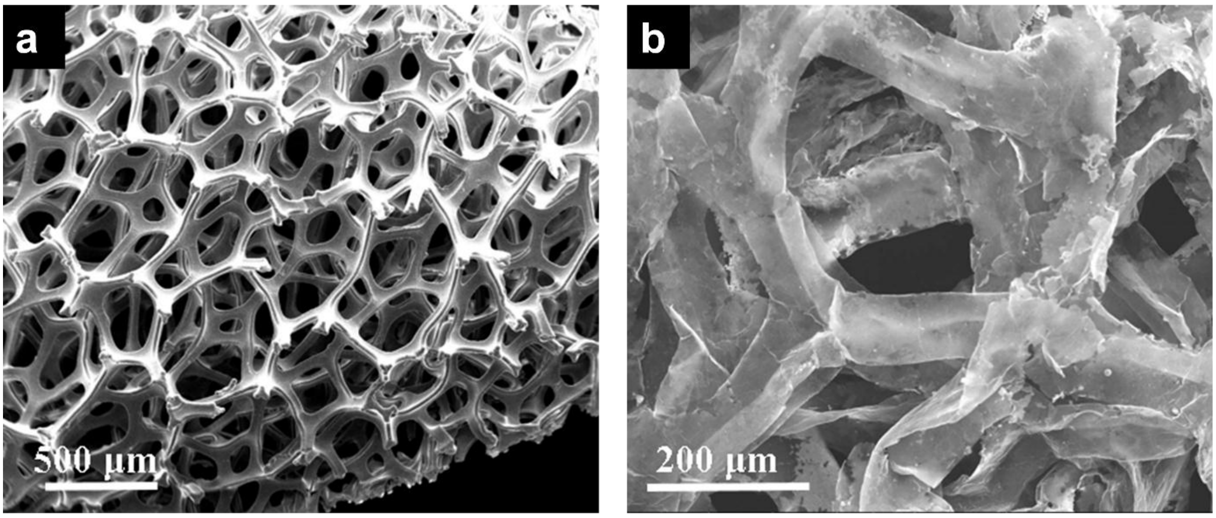


**Figure S14**. SEM images of (a) Ni/graphene foam, (b) graphene foam as negative electrode material in this study.

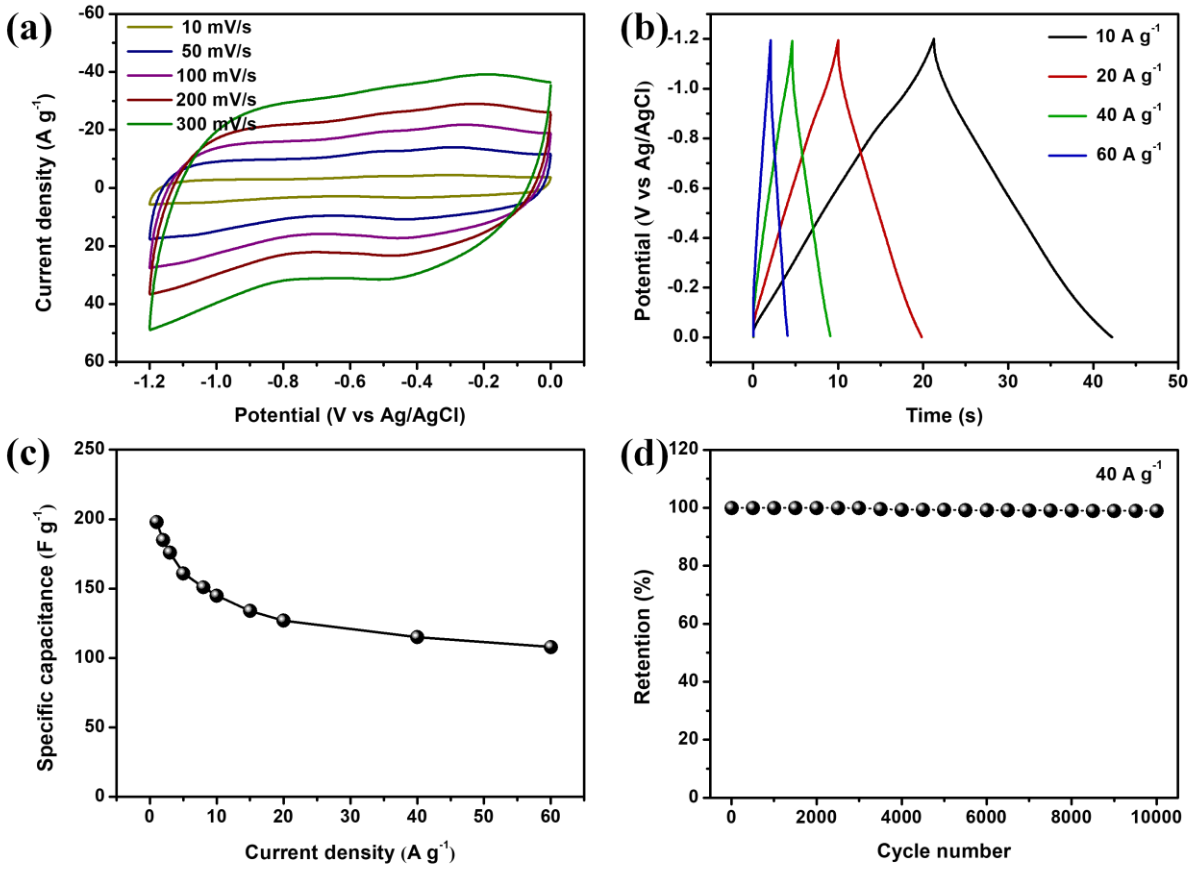


**Figure S15**. The electrochemical performance of the graphene foam negative electrode in 2 M KOH electrolyte. (a) CV, (b) GCD, (c) specific capacitance at different current densities, (d) cycling stability at a current density of 40 A $g^{-1}$ for 10 000 cycles.

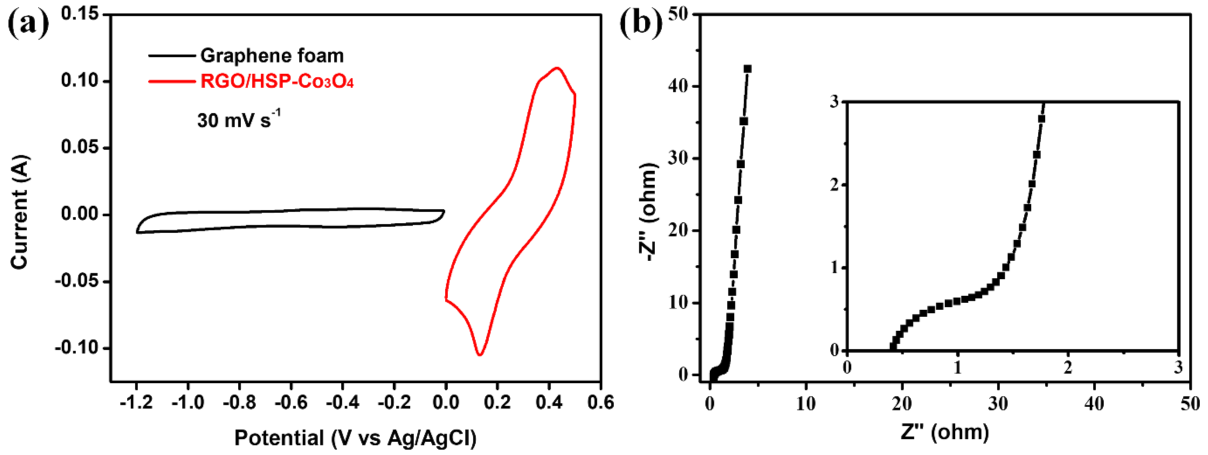


**Figure S16**. (a) The cyclic voltammetry curves of the RGO/HSP-$Co_3O_4$ electrode and graphene foam electrode at the same scan rate of 30 mV $s^{-1}$ in 2 M KOH electrolyte. (b) EIS curves of the ASC device.
